# PDZK1 and NHERF1 Regulate the Function of Human Organic Anion Transporting Polypeptide 1A2 (OATP1A2) by Modulating Its Subcellular Trafficking and Stability

**DOI:** 10.1371/journal.pone.0094712

**Published:** 2014-04-11

**Authors:** Jian Zheng, Ting Chan, Florence Shin Gee Cheung, Ling Zhu, Michael Murray, Fanfan Zhou

**Affiliations:** 1 Alkali Soil Natural Environmental Science Center, Northeast Forestry University/Key Laboratory of Saline-alkali Vegetation Ecology Restoration in Oil Field, Ministry of Education, Harbin, China; 2 Faculty of Pharmacy, The University of Sydney, Sydney, New South Wales, Australia; 3 Retinal Therapeutics Research Group, Save Sight Institute, The University of Sydney, Sydney, New South Wales, Australia; 4 Discipline of Pharmacology, School of Medical Sciences, The University of Sydney, Sydney, New South Wales, Australia; Harvard Medical School, United States of America

## Abstract

The human organic anion transporting polypeptide 1A2 (OATP1A2) is an important membrane protein that mediates the cellular influx of various substances including drugs. Previous studies have shown that PDZ-domain containing proteins, especially PDZK1 and NHERF1, regulate the function of related membrane transporters in other mammalian species. This study investigated the role of PDZK1 and NHERF1 in the regulation of OATP1A2 in an *in vitro* cell model. Transporter function and protein expression were assessed in OATP1A2-transfected HEK-293 cells that co-expressed PDZK1 or NHERF1. Substrate (estrone-3-sulfate) uptake by OATP1A2 was significantly increased to ∼1.6- (PDZK1) and ∼1.8- (NHERF1) fold of control; this was dependent on the putative PDZ-binding domain within the C-terminus of OATP1A2. The functional increase of OATP1A2 following PDZK1 or NHERF1 over-expression was associated with increased transporter expression at the plasma membrane and in the whole cell, and was reflected by an increase in the apparent maximal velocity of estrone-3-sulfate uptake (V_max_: 138.9±4.1 (PDZK1) and 181.4±16.7 (NHERF1) versus 55.5±3.2 pmol*(µg*4 min)^−1^ in control; P<0.01). Co-immunoprecipitation analysis indicated that the regulatory actions of PDZK1 and NHERF1 were mediated by direct interaction with OATP1A2 protein. In further experiments PDZK1 and NHERF1 modulated OATP1A2 expression by decreasing its internalization in a clathrin-dependent (but caveolin-independent) manner. Additionally, PDZK1 and NHERF1 enhanced the stability of OATP1A2 protein in HEK-293 cells. The present findings indicated that PDZK1 and NHERF1 regulate the transport function of OATP1A2 by modulating protein internalization via a clathrin-dependent pathway and by enhancing protein stability.

## Introduction

Organic anion transporting polypeptides (OATPs) encoded by solute carrier transporter (*SLC*) genes are a family of membrane transporter proteins that are widely expressed in human tissues. OATPs mediate the cellular influx of endogenous and exogenous substances including a number of drugs [1]–[7]. OATP1A2 is expressed in the renal tubules, intestine, brain capillary endothelium and biliary cholangiocytes where it contributes to intestinal drug absorption at the luminal apical membrane of enterocytes, the tubular reabsorption of xenobiotics at the renal apical membrane and drug transport into brain [3], . Important substrates of OATP1A2 include bile acids, steroid and thyroid hormones (and their conjugates), drugs such as imatinib, fexofenadine, methotrexate, HIV protease inhibitors and HMG-CoA reductase inhibitors, and certain peptides [2], [3], [11], [14]–[20]. Indeed, OATP1A2 function influences the cellular uptake and pharmacokinetic behaviour of a number of drugs [1], [3], [4], [13], [19].

Several important regulatory mechanisms have emerged for OATPs, including post-translational processing by N-glycosylation [21] and altered trafficking by protein kinase-mediated phosphorylation [22]–[25]. OATPs are also regulated by interactions with chaperones that include the PDZ (PSD95, D1g and ZO1) domain-containing proteins [26]–[28]. These proteins interact with small canonical sequences of 3–4 amino acid residues within C-termini of target proteins to direct and anchor the latter to specific regions of the cell [29]. The PDZ proteins PDZK1 and NHERF1 have been shown to regulate the subcellular trafficking of certain transporters and to be essential for optimal transport function [30]–[32]. PDZK1 regulates the cellular polarity of rodent Oatps by maintaining the asymmetrical distribution of proteins and lipids between the apical and basolateral surfaces of cells [27], [28]. Phosphorylation on serine residues upstream from the PDZ binding consensus site in rat Oatp1a1 is required for optimal binding to PDZK1 [26]. A recent study also implicated PDZK1 in the selective recruitment of microtubule-based motor proteins that mediate murine Oatp1a1 trafficking to the plasma membrane [33].

From yeast two-hybrid library screening Kato *et al*. first suggested the potential for interactions between PDZ proteins and human OATP1A2 [34]. The present study investigated the molecular mechanisms by which PDZ proteins regulate OATP1A2 transport function. The major findings to emerge were that PDZ proteins regulate OATP1A2 function and expression in HEK-293 cells by modulation of transporter trafficking and protein stability.

## Materials and Methods

### Materials

[^3^H]Estrone-3-sulfate (E3S; specific activity 57.3 Ci/mmol) was purchased from PerkinElmer (Melbourne, VIC, Australia). Dulbecco's modified Eagle's medium (DMEM) was obtained from Thermo Scientific (Lidcombe, NSW, Australia). Puromycin was purchased from Sapphire Biosciences (Redfern, NSW, Australia). Endo F and Endo Hf were purchased from Genesearch (Arundel, Qld, Australia). Protease inhibitor cocktail tablets were obtained from Roche Diagnostics Australia Pty. Ltd. (Castle Hill, NSW, Australia). Anti-flag and anti-myc antibodies were from Genesearch, the horseradish peroxidase-conjugated goat anti-rabbit IgG was from Sapphire Biosciences and the anti-β-actin antibody was obtained from Santa Cruz Biotechnology (Santa Cruz, CA, USA). Unless otherwise stated, all other chemicals and biochemicals were purchased from Sigma-Aldrich (Castle Hill, NSW, Australia).

### Preparation of OATP1A2, PDZK1 and NHERF1 constructs

The OATP1A2, PDZK1 and NHERF1 cDNAs were purchased from GeneCopoeia (Cat. No: GC-Q0577, GC-Q0230 and GC-U0225) and subcloned into the PCI vector (Promega; Alexandria, NSW, Australia). Nucleotide changes were generated in cDNAs by site-directed mutagenesis using Pfu DNA polymerase (Promega; Singapore) as described previously [35], [36]. A Flag tag (DYKDDDDK) was inserted at the N-terminus of OATP1A2 and myc tags (EQKLISEEDL) were introduced into the N-termini of PDZK1 and NHERF1. The OATP1A2-del mutant, that lacked the PDZ binding domain (residues 667–670, KTKL), was constructed by site-direct mutagenesis as described elsewhere [1], [37] with the forward primer: 5′- GAAAGATGATGAATTGTAGCGGTACCTCTAG-3′ and the reverse primer: 5′- CTAGAGGTACCGCTACAATTCATCATCTTTC-3′. All sequences were confirmed by the dideoxy chain termination method (Ramaciotti Centre, University of New South Wales, Randwick, NSW, Australia).

### Substrate uptake in HEK-293 cells that over-express OATP1A2

HEK-293 cells were cultured in DMEM supplemented with 10% fetal calf serum (37°C, 5% CO_2_). Cells were transfected with plasmid DNA using Lipofectamine 2000 (Invitrogen, Mount Waverley, VIC, Australia). Twenty-four h after transfection cellular uptake of [^3^H]-E3S (final concentration 0.3 µM, 67 nCi/well) was estimated as described previously [1], [2],[23],[38]. Uptake was initiated in phosphate-buffered saline (PBS; 137 mM NaCl, 2.7 mM KCl, 4.3 mM Na_2_HPO_4_, 1.4 mM KH_2_PO_4_, pH 7.4) containing 1 mM CaCl_2_, and 1 mM MgCl_2_. Preliminary experiments indicated that initial rates of OATP1A2-mediated substrate uptake in HEK-293 cells were linear over at least 10 mins. Uptake was terminated by rapidly washing the cells in PBS buffer at 4°C. The cells were then solubilized in 0.2 M NaOH, followed by neutralization with 0.2 M HCl. Uptake rates were standardized to the total amount of protein in each well. Kinetic studies were conducted over the E3S concentration range 0.05–50 µM and were run for 4 min. Apparent K_m_ and V_max_ values for substrate uptake were calculated by nonlinear regression (GraphPad Prism 5.0; GraphPad Inc, LaJolla, CA, USA).

### Electrophoresis and immunoblotting

Cell lysate proteins were denatured, loaded onto 7.5% polyacrylamide minigels and electrophoresed as described previously [1], [23], [37]. Protein transfer to polyvinylidene fluoride membranes was conducted in an electroelution cell (Bio-Rad; Gladesville, NSW, Australia). Membranes were blocked for 1 h with 5% nonfat dry milk in PBS-Tween (137 mM NaCl, 2.7 mM KCl, 4.3 mM Na_2_HPO_4_, 1.4 mM KH_2_PO_4_ and 0.05% Tween 20, pH 7.5), washed with TBS-Tween, and then incubated overnight at 4°C with primary antibody. Membranes were washed, incubated with horseradish peroxidase-conjugated goat anti-rabbit IgG (1∶5000; Sapphire Biosciences, Cat. No: sc-2004), and signals were detected using the Immobilon Western Chemiluminescent HRP Substrate (Merck; Kilsyth, VIC, Australia).

### Co-immunoprecipitation

HEK-293 cells were co-transfected with OATP1A2-N-flag and either PDZK1-N-myc or NHERF1-N-myc and were lysed 24 h later in gentle immunoprecipitation buffer (10 mm Tris/HCl, 10 mm NaCl, 0.5–1% Triton X-100, 2 mm EDTA, 10% glycerol and protease inhibitor cocktail, pH 7.5). Cell lysates were precleaned with protein G-agarose beads (Genesearch) to decrease nonspecific binding and then incubated overnight with anti-flag antibody (Genesearch; 1∶200) at 4°C. Protein G-agarose beads were then added and mixed by end-over-end rotation at 4°C for 2 h. Proteins bound to the protein G-agarose beads were eluted with Laemmli buffer containing β-mercaptoethanol and analyzed by immunoblotting with anti-myc antibody (Genesearch; 1∶1000).

### Biotinylation of OATP1A2 expressed at the plasma membrane of HEK-293 cells

HEK-293 cells were co-transfected with OATP1A2-N-flag and PDZK1 or NHERF1 cDNAs, or vector alone (control), in six-well plates using Lipofectamine 2000 [37]. After 24 h, the medium was removed and the cells were washed with ice-cold PBS (pH 8.0; 3 mL). Cells were incubated on ice with the membrane impermeable NHS-SS-biotin (0.5 mg/mL in PBS). After 30 min, cells were washed with PBS containing 100 mM glycine and incubated on ice for 20 min to ensure complete quenching of unreacted NHS-SS-biotin. The cells were then treated for 30 min with lysis buffer (10 mM Tris, 150 mM NaCl, 1 mM EDTA, 0.1% sodium dodecyl sulfate, 1% Triton X-100, that contained the protease inhibitors phenylmethylsulfonyl fluoride, 200 mg/mL, and leupeptin, 3 mg/mL, pH 7.4; 400 µL). Unlysed cells were removed by centrifugation at 14,000 g at 4°C.

Equivalent quantities of protein lysates from each sample (Bradford assay) were loaded onto streptavidin-agarose beads (50 µL; Quantum Scientific), eluted and subjected to immunoblotting analysis, as described above. After probing with the OATP1A2 or Flag antibodies, the membranes of biotinylated samples were routinely re-probed with anti-β-actin antibody to confirm the absence of the intracellular protein β-actin. In addition, 10% of each lysate was denatured and loaded onto separate gels. Immunoblotting for β-actin on the membranes of lysate was done to confirm uniform protein loading.

### OATP1A2 internalization

**Figure 1:**
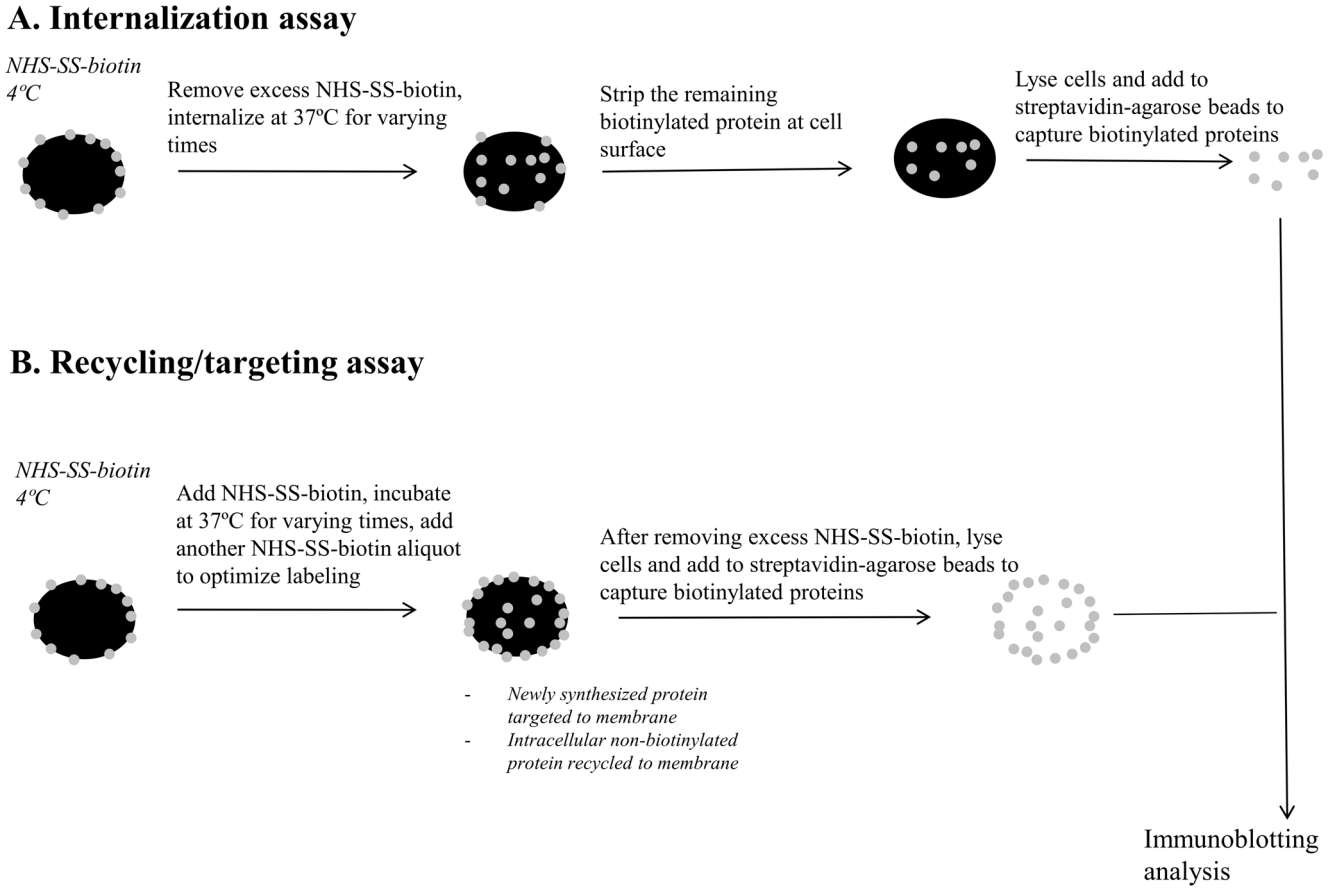
Scheme outlining the biotinylation-based internalization and recycling/membrane targeting assays. (A) Internalization assay. HEK-293 cells co-transfected with OATP1A2-N-flag and PDZK1-N-myc, NHERF1-N-myc or control vector were labeled with NHS-SS-biotin at 4°C. After quenching of residual NHS-SS-biotin with glycine (100 mM) the cells were warmed to 37°C to initialize internalization. At the end of incubations, sodium 2-mercaptoethanesulfonate (50 mM) was added to strip biotinylated, but non-internalized, proteins remaining at the cell surface. Cell lysates were prepared in lysis buffer and 500 µg protein from each sample was applied to streptavidin-agarose beads to capture biotinylated proteins. (B) Recycling/membrane targeting assay. HEK-293 cells co-transfected with OATP1A2-N-flag and PDZK1-N-myc, NHERF1-N-myc or control vector were labeled with NHS-SS-biotin at 4°C and then warmed to 37°C to initialize recycling/targeting for varying times; an additional aliquot of NHS-SS-biotin was applied to cells to optimize biotinylation of recycled/targeted proteins. At the end of incubations residual NHS-SS-biotin was quenched with glycine (100 mM) at 4°C. Cell lysates were prepared in lysis buffer and 500 µg protein from each sample was applied to streptavidin-agarose beads to capture biotinylated proteins. Electrophoresis and immunoblotting of lysate proteins bound to streptavidin/agarose beads was performed as described in Materials and Methods.

As shown in Fig 1A, internalization of biotinylated OATP1A2 was evaluated in HEK-293 cells that were co-transfected with OATP1A2-N-flag and PDZK1-N-myc, NHERF1-N-myc or control vector [23], [39]. Residual NHS-SS-biotin was quenched with glycine (100 mM) and the cells were warmed to 37°C to initialize internalization. At the end of incubations, sodium 2-mercaptoethanesulfonate (50 mM) in NT buffer (150 mM NaCl, 1 mM EDTA, 0.2% bovine serum albumin, 20 mM Tris, pH 8.6; 30 min, repeated twice) was added to strip biotinylated, but non-internalized, proteins remaining at the cell surface. Cell lysates were prepared in lysis buffer and 500 µg protein from each sample was applied to streptavidin-agarose beads to capture biotinylated proteins. Electrophoresis and immunoblotting of the lysate proteins bound to streptavidin-agarose beads was conducted as described above. Again uniform protein loading was confirmed by immunoblotting for β-actin in separate lysate aliquots. Densitometric analysis (ImageJ software) was used to estimate relative OATP1A2 internalization at each time point, which was expressed as a percentage of the initial biotinylated pool of OATP1A2 at the plasma membrane.

### OATP1A2 targeting/recycling

HEK-293 cells co-transfected with OATP1A2-N-flag and PDZK1-N-myc, NHERF1-N-myc or control vector were labeled with NHS-SS-biotin (1 mg/mL) at 4°C and then warmed to 37°C to initialize recycling/targeting for varying times; an additional aliquot of NHS-SS-biotin (1 mg/mL, 30 min, 4°C) was applied to cells to optimize biotinylation of recycled/targeted proteins (Fig 1B). At the end of incubations residual NHS-SS-biotin was quenched with glycine (100 mM) at 4°C [23], [39]. Cell lysates were prepared in lysis buffer and 500 µg protein each sample was applied to streptavidin-agarose beads to capture biotinylated proteins. Electrophoresis and immunoblotting of the lysate proteins bound to streptavidin-agarose beads was conducted as described above. Again uniform protein loading was confirmed by immunoblotting for β-actin in separate lysate aliquots. Densitometric analysis (ImageJ software) was used to estimate the relative amount of recycled and membrane targeted OATP1A2 at each time point, which was expressed as a percentage of the initial biotinylated pool of OATP1A2 at the plasma membrane.

### Statistics

Data are presented throughout as mean±S.E. The Student's *t*-test was used to test for differences between two sets of normally distributed data and one-way analysis of variance and Dunnett's testing for multiple treatment comparisons.

## Results

### PDZK1 and NHERF1 enhance the expression and functional activity of OATP1A2 in HEK-293 cells

**Figure 2:**
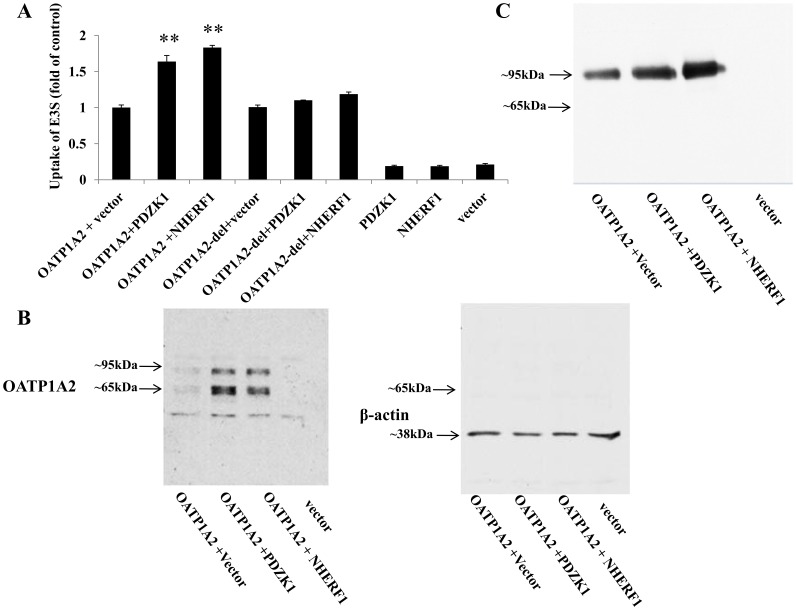
Altered transport function and expression of OATP1A2 in HEK-293 cells in the presence and absence of co-transfected PDZK1 and NHERF1. (A) Transport of 300 nM [^3^H] E3S in OATP1A2-transfected HEK-293 cells with or without co-expressed PDZK1 or NHERF1 at 37°C. Values are means ± S.E. (*n* = 3). **: Different from control (OATP1A2 + vector): *P*<0.01. (B) Western blot analysis of total cellular expression of OATP1A2-N-flag isoforms with or without co-expression of PDZK1 or NHERF1. *Top Panel*: Cells were lysed and proteins were separated by SDS-polyacrylamide gel electrophoresis, followed by Western blotting with anti-flag antibody. *Bottom Panel*: After stripping, the blot was reprobed with anti-β-actin antibody. (C) Western blot analysis of cell surface expression of OATP1A2-N-flag with or without co-expression of PDZK1 or NHERF1. Cells were biotinylated, and the labeled cell surface proteins were precipitated with streptavidin beads and separated by gel electrophoresis, followed by Western blotting with anti-flag antibody.

The interaction between PDZ proteins and human OATP1A2 was identified by yeast two-hybrid library screening [34], but the functional significance has not been established. In the current study, we tested whether PDZ proteins alter transporter function after co-expression in HEK-293 cells. As shown in Fig. 2A, co-expression of the major PDZ proteins, PDZK1 and NHERF1, enhanced the transporter activity of OATP1A2 in HEK-293 cells to ∼1.6- (PDZK1) and ∼1.8- (NHERF1) fold of control (OATP1A2 + vector). Because the available antibodies of OATP1A2, PDZK1 and NHERF1 lack specificity, we prepared N-Flag tagged OATP1A2 and N-myc tagged PDZK1 and NHERF1 constructs for use in this study. This enabled the use of the highly specific anti-flag and anti-myc antibodies in the analysis of OATP1A2/PDZ protein interactions. Inclusion of the Flag and myc tags at the N-terminus did not influence OATP1A2 function or its activation by PDZK1 or NHERF1 (data not shown).

The putative PDZ binding domain of OATP1A2 is located at the last four C-terminal amino acids of the transporter (residues 667–670, KTKL); this motif is also highly conserved among OATPs. An OATP1A2-del mutant cDNA that lacked the PDZ binding domain was constructed by site-directed mutagenesis. In functional experiments neither PDZK1 nor NHERF1 enhanced E3S uptake by the OATP1A2-del mutant, although basal uptake of this mutant was comparable to wild type OATP1A2 (Fig 2A).

Consistent with previous findings [1], two signals were observed at ∼95 KDa and ∼65 KDa in direct immunoblots of OATP1A2-N-flag in total cell lysates from HEK-293 cells (Fig 2B). In contrast, only the ∼95 KDa isoform was detected on immunoblots after biotinylation (Fig 2C), which supports the contention that this isoform may be a fully glycosylated mature surface form of OATP1A2, while the ∼65 KDa isoform may be an immature intracellular form. In supporting experiments, both OATP1A2 isoforms were sensitive to treatment with Endo F (an endoglycosidase that cleaves N-linked oligosaccharides in glycoproteins), but only the ∼65 KDa isoform exhibited a band shift after treatment with Endo Hf (an endoglycosidase that cleaves high mannose and some hybrid oligosaccharides from *N*-linked glycoproteins) (data not shown).

**Figure 3:**
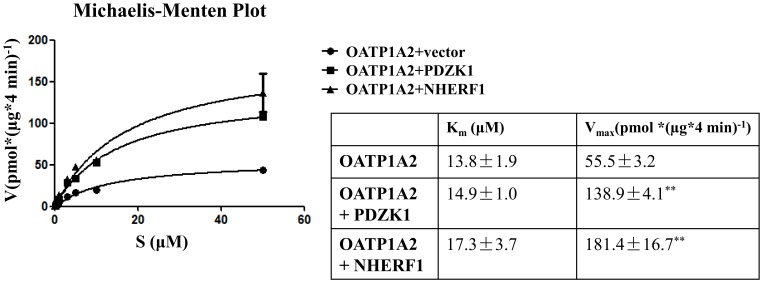
Michaelis-Menten plot of E3S transport kinetics by OATP1A2 with or without co-transfected PDZK1 or NHERF1. E3S uptake was conducted over a 4-specific uptake by vector transfected cells and was also standardized to the amount of protein in each well. Kinetic parameters were calculated by nonlinear regression. Values are means ± S.E. (n = 3). **: Different from control: *P*<0.01.

Co-expression of PDZK1 or NHERF1 also increased plasma membrane expression of OATP1A2 to ∼1.7±0.3- (PDZK1) and ∼2.0±0.5- (NHERF1) fold of control (n = 3; Fig 2C) and total cellular expression of the ∼95 KDa and ∼65 KDa isoforms (P<0.01, Fig 2B). These findings were supported by the kinetic analysis that found the V_max_, but not the K_m_, for E3S uptake by OATP1A2 was increased by co-expression of PDZ proteins (P<0.01; Fig 3).

### PDZK1 and NHERF1 interact with OATP1A2

**Figure 4:**
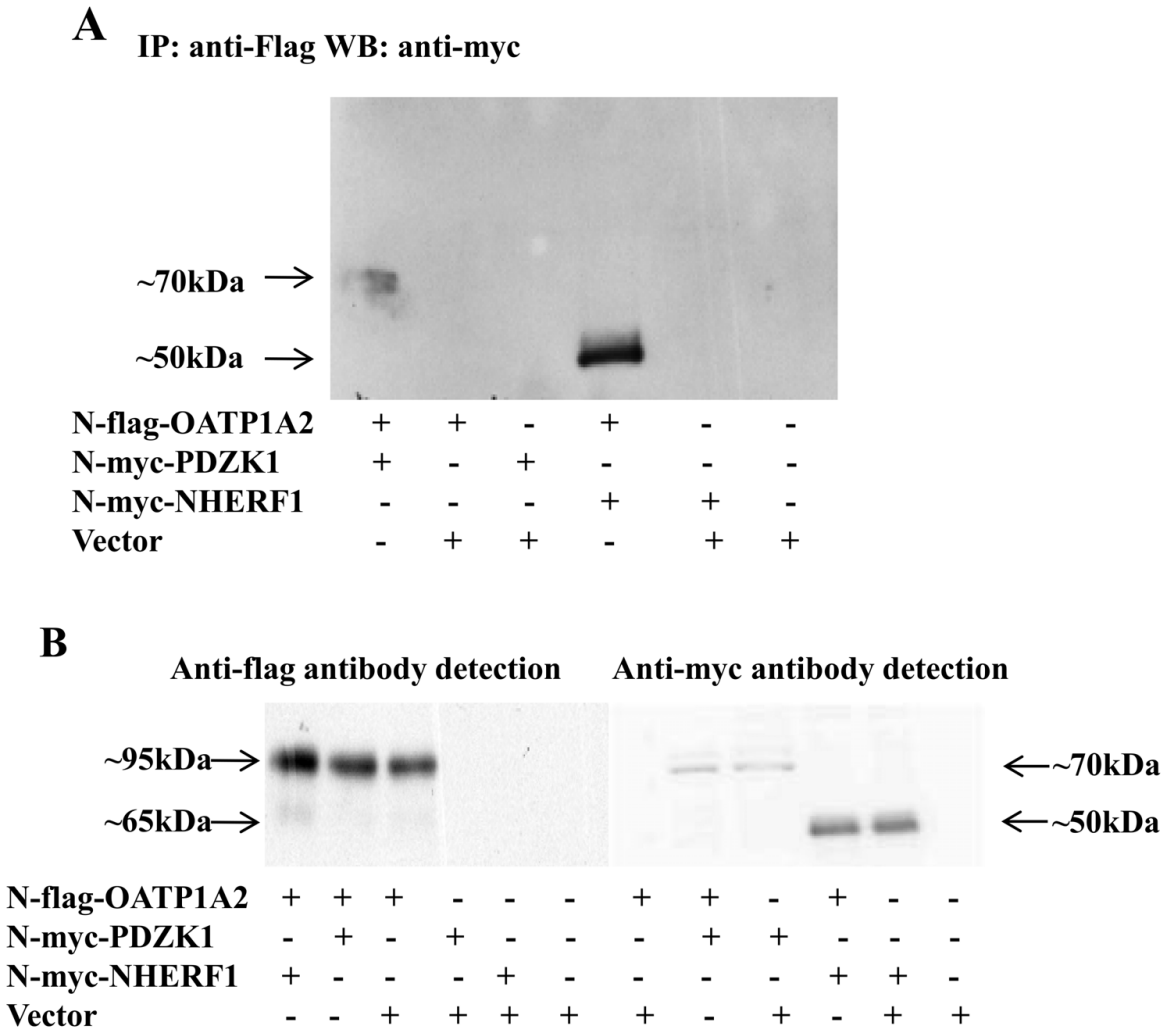
Association of OATP1A2 with PDZK1 and NHERF1. (A) co-immunoprecipitation of OATP1A2-N-flag and PDZK1-N-myc or NHERF1-N-myc. HEK-293 cells that co-expressed OATP1A2-N-flag and either PDZK1-N-myc or NHERF1-N-myc were lysed and subjected to immunoprecipitation with anti-flag antibody, followed by immunoblotting with anti-myc antibody. (B) Direct immunoblotting for N-Flag and N-myc tags, respectively, in the lysates of HEK-293 cells co-expressed with OATP1A2-N-flag and either PDZK1-N-myc or NHERF1-N-myc prior to immunoprecipitation.

PDZK1-N-myc or NHERF1-N-myc constructs were transiently co-transfected with the OATP1A2-N-flag construct into the HEK-293 cells. Cell lysates were prepared, immune precipitates were isolated with the anti-flag antibody, and were then subjected to immunoblotting for myc-tagged PDZK1 or NHERF1. As shown in Fig 4A, specific bands were detected in immune precipitates at ∼70 KDa (PDZK1) and ∼50 KDa (NHERF1), which correspond to the anticipated sizes of PDZK1 and NHERF1 [30], [40]. The findings in Figs 4B and 4C were obtained by direct immunoblotting of cell lysates with anti-flag and anti-myc, respectively. Together these findings suggest that OATP1A2 interacts directly with PDZ proteins.

### PDZK1 and NHERF1 regulate subcellular trafficking of OATP1A2

**Figure 5:**
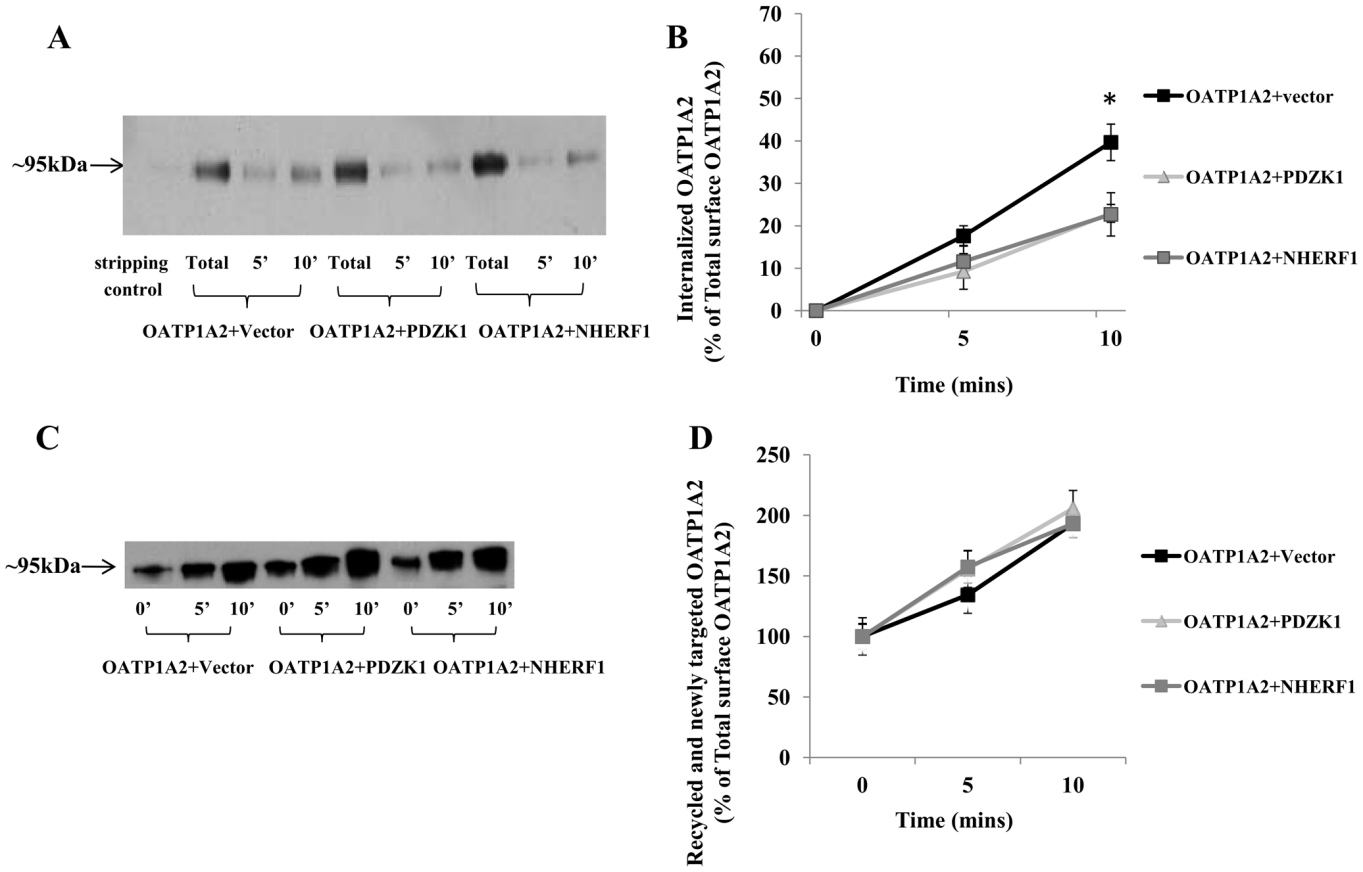
Biotinylation analysis of OATP1A2-N-flag internalization and recycling/targeting in HEK-293 cells co-expressing PDZK1-N-myc and NHERF1-N-myc. (A) OATP1A2-N-flag internalization was undertaken as described under “Materials and Methods,” followed by Western blotting with an anti-Flag antibody; a representative of 3 separate experiments was shown. *Stripping control*: biotinylated cells were treated with 50 mM sodium 2-mercaptoethanesulfonate to remove the biotin label prior to initiating internalization. (B) Densitometric analysis of internalized OATP1A2-N-Flag as a percentage of the total initial pool of biotinylated OATP1A2-N-Flag at the cell surface (means ±S.E. from 3 individual experiments). *: Different from control: *P*<0.05. (C) OATP1A2-N-Flag recycling/targeting was undertaken as described under “Materials and Methods,” followed by Western blotting with an anti-Flag antibody; a representative of 3 separate experiments was shown. (D) Densitometric analysis of membrane OATP1A2-N-Flag as a percentage of the pool of biotinylated OATP1A2-N-Flag prior to initiation of recycling/targeting (means ±S.E. from 3 individual experiments). Blotting for β-actin in separate aliquots of total lysates was used to confirm uniform protein loading.

The increased cell surface expression of OATP1A2 following co-expression of PDZK1 or NHERF1 could be due to decreased protein internalization (endocytosis), increased recycling/targeting between the plasma membrane and intracellular compartments, or a combination of both. As shown previously, OATP1A2 is subject to constitutive internalization [23]. In the present studies OATP1A2-N-flag was co-expressed with PDZK1 or NHERF1. Cell surface proteins were pre-labeled with membrane impermeable NHS-SS-biotin and then subjected to experimental protocols to individually evaluate OATP1A2 internalization and recycling/targeting (Fig 1). As shown in Fig. 5A and 5B, the internalization rate of OATP1A2 was significantly decreased by co-expression of PDZK1 and NHERF1 (P<0.05). In contrast with this finding, PDZK1 and NHERF1 did not alter OATP1A2 trafficking from intracellular compartments to the cell membrane (Fig 5C and 5D). These findings suggest that OATP1A2 internalization, but not recycling/targeting, is regulated by PDZ proteins.

### PDZK1 and NHERF1 modulate OATP1A2 internalization via a clathrin-dependent pathway

Membrane transporter internalization may be clathrin-dependent, caveolin-dependent or clathrin/caveolin-independent. Here protein is relocated from the cell membrane to the cytoplasm via clathrin-coated pits and/or caveolin-enriched membrane invaginations [41], [42]. Previous studies have shown that the internalization of OATP1A2 and OATP2B1 is associated with clathrin-dependent pathways in renal cells *in vitro* [22], [23], while certain other proteins, such as the norepinephrine transporter in human placental trophoblast cells, are subject to caveolin-dependent endocytosis [43]. In the current study, inhibitors of clathrin- or caveolin-dependent pathways were used to evaluate OATP1A2 internalization in HEK-293 cells. Previous studies from this and other laboratories have indicated that clathrin-dependent endocytosis is effectively inhibited by K^+^-depletion or acidification of the cytoplasm [23], [39], [44], whereas caveolin-dependent endocytosis is prevented by filipin [22], [23], [45], [46] and nystatin [22], [23], [47].

**Figure 6:**
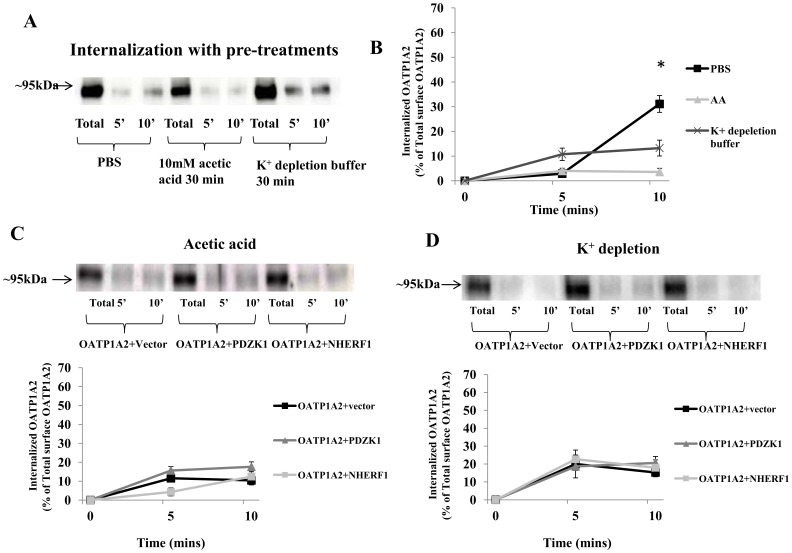
Clathrin-dependent internalization of OATP1A2 by PDZK1 and NHERF1. (A) Disruption of the clathrin-dependent pathway impairs OATP1A2-N-flag internalization. HEK-293 cells over-expressing OATP1A2-N-flag were treated with K^+^ depletion buffer or 10 mM acetic acid for 30 mins, relative to PBS-treated control. Internalization continued at 37°C for 5 or 10 mins as described under “Materials and Methods,” followed by immunoblotting for OATP1A2-N-flag. (B) Internalized OATP1A2-N-flag as a percentage of the total initial pool of biotinylated OATP1A2-N-flag at the cell surface (means±S.E. of 3 individual experiments). *: Different from control: *P*<0.05. (C) *Top panel*: immunoblot analysis of OATP1A2-N-flag in HEK-293 cells containing co-expressed PDZK1-N-myc or NHERF1-N-myc after treatment with 10 mM acetic acid for 30 mins, followed by internalization at 37°C for 5 or 10 mins. *Bottom panel*: Internalized OATP1A2-N-flag as a percentage of the total initial pool of biotinylated OATP1A2-N-flag at the cell surface (means±S.E. of 3 individual experiments). (D) *Top panel*: immunoblot analysis of OATP1A2-N-flag in HEK-293 cells containing co-expressed PDZK1-N-myc or NHERF1-N-myc after treatment with K^+^ depletion buffer for 30 mins, followed by internalization at 37°C for 5 or 10 mins. *Bottom panel*: Internalized OATP1A2-N-flag as a percentage of the total initial pool of biotinylated OATP1A2-N-flag at the cell surface (means±S.E. of 3 individual experiments). Blotting for β-actin in separate aliquots of total lysates was used to confirm uniform protein loading.

**Figure 7:**
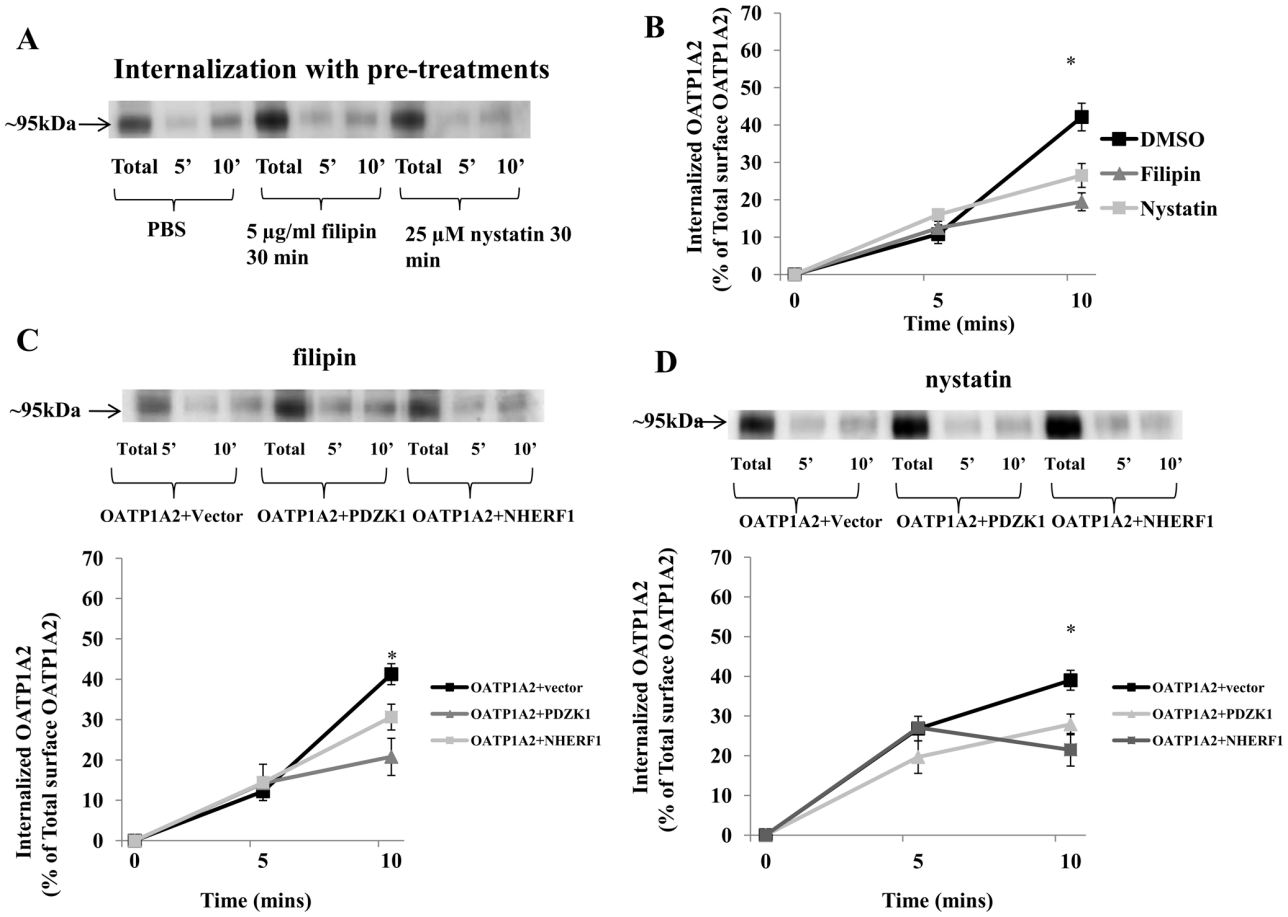
Caveolin-dependent internalization of OATP1A2 by PDZK1 and NHERF1. (A) Disruption of the caveolin-dependent pathway impairs OATP1A2-N-flag internalization. HEK-293 cells over-expressing OATP1A2-N-flag were treated with filipin (5 µg/ml) or nystatin (25 µM) for 30 mins, relative to dimethylsulfoxide-treated control. The cells were allowed to internalize at 37°C for 5 or 10 mins as described under “Materials and Methods” followed by immunoblotting for OATP1A2-N-flag. (B) Internalized OATP1A2-N-flag as a percentage of the total initial pool of biotinylated OATP1A2-N-flag at the cell surface (means±S.E. of 3 individual experiments) *: Different from control: *P*<0.05. (C) *Top panel*: immunoblot analysis of OATP1A2-N-flag in HEK-293 cells containing co-expressed PDZK1-N-myc or NHERF1-N-myc after treatment with filipin (5 µg/ml) for 30 mins, followed by internalization at 37°C for 5 or 10 mins. *Bottom panel*: Internalized OATP1A2-N-flag as a percentage of the total initial pool of biotinylated OATP1A2-N-flag at the cell surface (means±S.E. of 3 individual experiments). *: Different from control: *P*<0.05 (D) *Top panel*: immunoblot analysis of OATP1A2-N-flag in HEK-293 cells containing co-expressed PDZK1-N-myc or NHERF1-N-myc after treatment with nystatin (25 µM) for 30 mins, followed by internalization at 37°C for 5 or 10 mins. *Bottom panel*: Internalized OATP1A2-N-flag as a percentage of the total initial pool of biotinylated OATP1A2-N-flag at the cell surface (means±S.E. of 3 individual experiments). *: Different from control: *P*<0.05. Blotting for β-actin in separate aliquots of total lysates was used to confirm uniform protein loading.

The present data suggest that the constitutive internalization of OATP1A2 in HEK-293 cells is both clathrin- (Fig 6A and 6B) and caveolin- (Fig 7A and 7B) dependent. Interestingly, the decreased OATP1A2 internalization following the co-expression of PDZK1 or NHERF1 was diminished by pre-treatment with either acetic acid or K^+^-depletion buffer (Fig 6C and 6D), but not by pre-treatment with filipin or nystatin (Fig 7C and 7D).

### PDZK1 and NHERF1 regulate OATP1A2 expression by increasing protein stability

**Figure 8:**
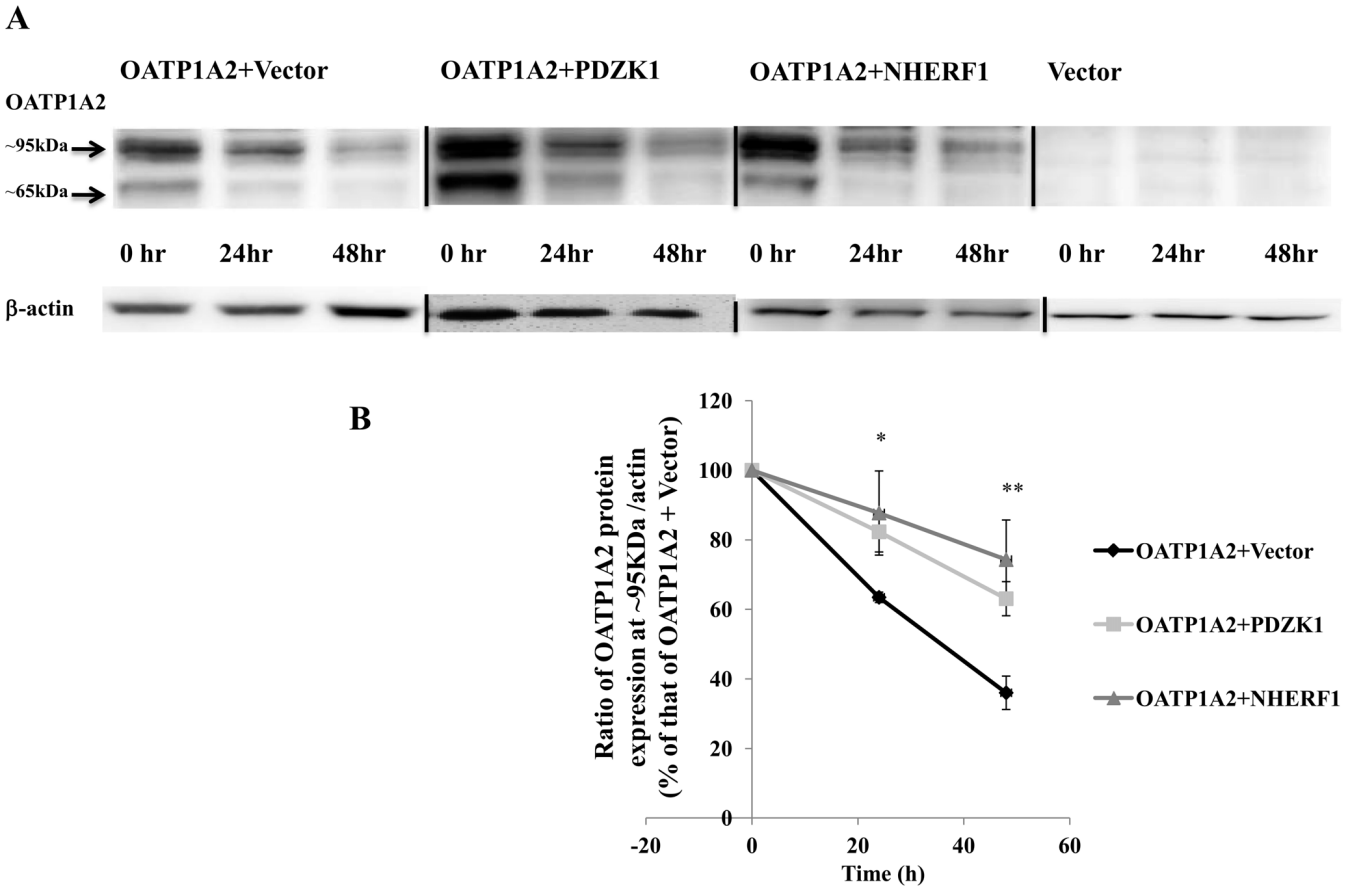
Stability of OATP1A2 protein in HEK-293 cells in the presence or absence of co-expressed PDZK1 or NHERF1. (A) Western analysis of total cellular expression of OATP1A2-N-flag with or without co-expression of PDZK1 or NHERF1. *Top Panel*: HEK-293 cells were treated with 5 µg/ml puromycin for 24 and 48 h. Cells were harvested and lysate proteins were separated by SDS-polyacrylamide gel electrophoresis, followed by Western blotting with anti-flag antibody. *Bottom Panel*: After stripping, blots were reprobed with anti-β-actin antibody. (B) Densitometric analysis of the mature (∼95 KDa) isoform of OATP1A2-N-flag as a percentage of total OATP1A2 protein in the absence of puromycin treatment (means±S.E. of 3 individual experiments). Different from control: *P<0.05; **P<0.01.

As shown in Fig. 2C, the co-expression of OATP1A2 with PDZK1 and NHERF1 increased the total cellular expression of the transporter. From real-time PCR analysis, this was not due to pre-translational up-regulation (data not shown). In further studies the stability of OAT1A2 protein was assessed in HEK-293 cells that had been treated with puromycin, an inhibitor of *de novo* protein synthesis [48], [49]. It emerged that the degradation rate of the ∼95 KDa isoform of OATP1A2 was significantly decreased by co-expression of PDZK1 and NHERF1 under these conditions (Fig 8A and 8B).

## Discussion

OATP1A2 facilitates the cellular uptake of hormones and other endogenous substrates, and drugs and other exogenous compounds. OATP1A2 is expressed in cells of kidney, intestine, cholangiocytes and brain and is an important determinant of drug penetration into those tissues [8], [11]–[13]. Currently, there is only limited information regarding the mechanisms that regulate OATP1A2 function; such information could have clinical and physiological significance.

There are about 180 human PDZ-domain-containing proteins. The mechanisms by which PDZ protein-protein interactions regulate the biological functions of proteins include altered phosphorylation, autoinhibition and allostery [50]. PDZ protein-protein interactions regulate membrane transporters in polarized epithelial cells [26]–[28], [30]–[32], [40], [51]–[54]. Interactions between PDZ proteins and SLC transporters, including OATP1A2, were first identified by Kato *et al*. using yeast two-hybrid screening [34]. Subsequent studies confirmed that interactions between PDZK1 and Oatp1a maintained the cellular polarity of murine Oatp1a [27], [28]. To our knowledge the present study is the first to report the interaction between OATP and NHERF1. Due to the intermolecular association between NHERF1 and PDZK1 [55], the present findings suggest that PDZ proteins form complexes that modulate the expression and function of OATP1A2. The present study provides novel mechanistic detail underlying the regulation of human OATP1A2 by PDZ proteins. Increased OATP1A2 function was reflected by the increase in protein expression at the plasma membrane and in the whole cell (Fig 2B, 2C and 2D) and by the increased V_max_ for E3S uptake (Fig 3).

PDZ proteins may regulate transporter functions by several mechanisms. PDZK1 and NHERF1 modulate the function of human organic anion transporter 4 (OAT4) by inhibiting internalization, which increases expression at the plasma membrane [30], [51]. Increased transporter function and cell surface expression following co-expression of PDZ proteins in cells has also observed for human proton-coupled peptide transporter 2 (hPepT2) and Organic Cation/Ergothioneine Transporter 1 (OCTN1) [31], [40], [52]. PDZ proteins also stabilized the apical expression of human organic cation/carnitine transporter 2 (OCTN2) [53], [54]. PDZ-dependent modulation of the cellular polarity of rat Oatp1a also required the phosphorylation of upstream serine residues [26]. The present data indicate that the increase in OATP1A2 function that occurred with co-expression of PDZK1 and NHERF1 is dependent on the PDZ binding domain located within the C-terminus of the transporter (residues 667–670, KTKL); this motif is highly conserved among OATPs.

It is well established that membrane transporters follow a constitutive internalization/recycling process in which they shuttle between the cell surface and intracellular compartments. Expression at the plasma membrane reflects both newly synthesized protein that is specifically targeted to the cell surface and recycled protein. During internalization, protein is relocated from the cell membrane to the cytoplasm by associating with clathrin-coated pits and/or caveolin-enriched membrane invaginations [41], [42]. Internalization of OATP1A2 in HEK-293 cells is partially dependent on clathrin-dependent endocytosis; this process is regulated by PKC [23]. In the current study, it was found that increased surface expression of OATP1A2 with co-expressed PDZK1 and NHERF1 was due in part to decreased protein internalization (Fig 5A, 5B) but not to altered recycling/targeting (Fig 5C, 5D). In subsequent experiments both clathrin- and caveolin-dependent pathways were implicated in the constitutive internalization of OATP1A2 (Fig. 6A, 7A). It is noteworthy, however, that decreased internalization in the presence of PDZ proteins was clathrin-dependent (Fig 6C, 6D), but not caveolin-dependent (Fig 7C, 7D).

OATP1A2 degradation was assessed in the presence and absence of the protein synthesis inhibitor puromycin. The apparent half life of OATP1A2 immunoreactive protein in HEK 293 cells was ∼24 h under these conditions and was prolonged on co-expression of PDZK1 and NHERF1, a finding consistent with enhanced stability; OATP1A2 mRNA expression was unchanged. Interestingly, and consistent with the present findings regarding OATP1A2, PDZK1 has also been shown to enhance the stability of the scavenger receptor class B type I protein in mouse hepatocytes [56].

## Conclusion

In summary, the present findings indicate that (i) co-expression of PDZK1 and NHERF1 activates OATP1A2 function by direct interaction with the putative PDZ binding domain located within the C-terminus of the transporter; (ii) enhanced OATP1A2 function is due to increased protein expression at the plasma membrane following decreased clathrin-dependent protein internalization; and (iii) PDZ proteins also stabilize OATP1A2 protein expression in HEK 293 cells. The potential significance of the present findings is that PDZ proteins, in particular PDZK1 and NHERF1, may represent novel molecular targets for the modulation of the intracellular distribution of drugs that are transported by OATP1A2.
